# PET–CT for visualizing the pathophysiology of COPD in patients with early‐stage NSCLC

**DOI:** 10.1111/1759-7714.15474

**Published:** 2024-10-24

**Authors:** Haruki Kobayashi, Tateaki Naito

**Affiliations:** ^1^ Division of Thoracic Oncology Shizuoka Cancer Center Shizuoka Japan

## BACKGROUND

In the clinical management of lung cancer, the focus is predominantly on tumor detection and staging, and other coexisting are often overlooked. Chronic obstructive pulmonary disease (COPD) is a common comorbidity; however, it is rarely considered when interpreting positron emission tomography–computed tomography (PET–CT) imaging.

## OBJECTIVE

Herein, we present PET–CT images that clearly depict the features of accessory respiratory muscles in patients with COPD.

## CASE REPORT

**FIGURE 1:**
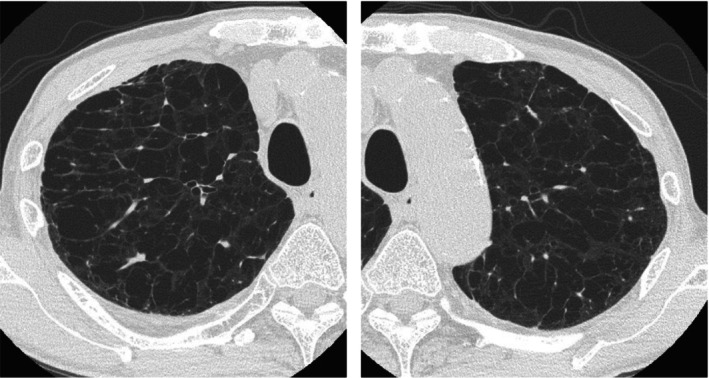
The axial view of the chest computed tomography (CT) scan acquired with a 1‐mm slice thickness at 120 kVp and 350 ms revealed severe emphysema.

A 74‐year‐old man with COPD (Figure 1) and no history of diabetes was referred to our hospital for suspected right‐lower‐lobe stage 1 lung cancer. Although long‐acting beta agonist/long‐acting muscarinic antagonist inhalation therapy was initiated for COPD, the patient experienced significant shortness of breath even during conversations held while sitting. Over the past 6 months, he experienced substantial weight loss (~10 kg) due to muscle atrophy, resulting in his body mass index decreasing to 16. The patient opted for exclusive best‐supportive care for stage 1 lung cancer owing to a performance status of 3.

**FIGURE 2:**
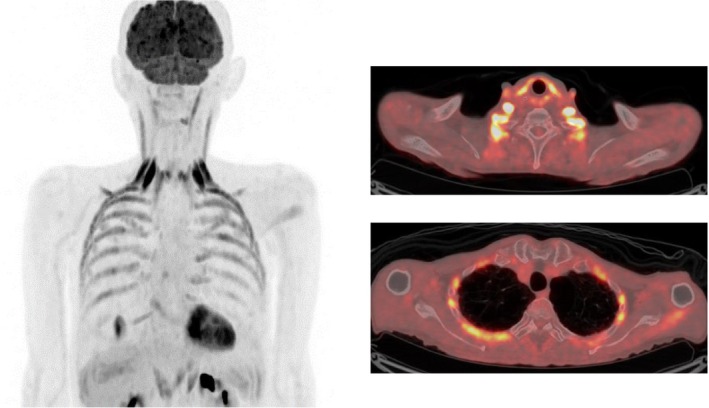
The 3D maximum intensity projection and fused positron emission tomography–computed tomography revealed significant ^18^F‐FDG uptake in the sternocleidomastoid and upper intercostal muscles and weak uptake in the diaphragm and lower intercostal muscles. Acquired at 120 kVp and 500 ms.

A PET–CT scan revealed strong uptake not only in the right‐lower‐lobe nodule but also in the sternocleidomastoid and upper intercostal muscles and weak uptake in the diaphragm and lower intercostal muscles (Figure 2).

## DISCUSSION

Herein, we present a case in which PET performed for lung cancer evaluation^1^ clearly demonstrated FDG uptake in the accessory respiratory muscles, a finding that has been previously reported.^2^

Patients with COPD often experience skeletal muscle atrophy, which can lead to reduced oxidative capacity and muscle weakening due to the presence of a higher proportion of type 2 muscle fibers compared with type 1 fibers.^3^ However, this shift toward type 1 fibers is observed in accessory respiratory muscles such as the intercostal muscles.^4^ The sternocleidomastoid and upper intercostal muscles also play a crucial role in daily respiratory function, unlike the diaphragm and lower intercostal muscles that are affected by airflow limitation.^5^ Therefore, the different strength of uptake by accessory respiratory muscles revealed by PET–CT highlights the muscle pathophysiology of patients with COPD.

## FUNDING INFORMATION

This report did not receive any specific grants from any funding agencies in the public, commercial, or not‐for‐profit sectors.

## CONFLICT OF INTEREST STATEMENT

The authors declare no conflicts of interest.

## Data Availability

The data that support the findings of this study are available on request from the corresponding author.
